# The meaning of ethical collaborative research according to young people

**DOI:** 10.1080/02673843.2025.2517099

**Published:** 2025-06-18

**Authors:** Rabab Chrifou, Farah Focquaert, Katrina Messiha, Mohammed Ghaly, Teatske Altenburg, Benedicte Deforche, Maïté Verloigne

**Affiliations:** a Department of Public Health and Primary Care, Ghent University, Ghent, Belgium; b Department of Philosophy and Moral Sciences, Faculty of Arts and Philosophy, Ghent University, Ghent, Belgium; c Amsterdam UMC Location Vrije Universiteit Amsterdam, Department of Public and Occupational Health, Amsterdam, The Netherlands; d Health Behaviours and Chronic Disease, Methodology, Amsterdam Public Health, Amsterdam, The Netherlands; e Research Centre for Islamic Legislation & Ethics, Hamad Bin Khalifa University, Doha, Qatar; f Movement and Nutrition for Health and Performance Research Group, Faculty of Physical Education and Physical Therapy, Vrije Universiteit Brussel, Brussels, Belgium

**Keywords:** Ethics, moral reasoning, collaborative research, co-creation, meta-moral cognition, youth

## Abstract

Incorporating the perspectives of young people regarding their perspectives on ethical collaborative research may enrich our current understanding of ethical practices within such research, as young people are capable of providing creative, innovative and novel perspectives that otherwise remain unknown to the adult academic establishment. Youth, Ethics & Participation (YEP), a qualitative study conducted as part of the Health CASCADE project, aimed at engaging young people around the nature and meaning of ethics within collaborative research while developing moral awareness. Young people shared their perspectives on the meaning of ethical collaborative research, while showing their interest and ability of moral reasoning through the formulation of arguments explaining their position on a variety of ethically salient issues within collaborative research. The current study indicates that the engagement of young people in collaborative research is a potential source for instigating social change through their engagement in moral reasoning.

## Introduction

Adult researchers are increasingly integrating the perspectives of young people regarding issues that affect their lives, while seeking an enhanced understanding of their worldviews (Coyne & Carter, 2018). Acknowledging young people as social actors in their own right, with a valuable and important voice of their own, is believed to contribute to societal well-being in both the short and long term (Alves et al., 2022; Patton et al., 2016). Moreover, engaging young people is increasingly viewed as an ethical practice, as their status as rights-bearing participants within both society and research is well established (Bell, 2008). Collaborative approaches in research (Loveridge et al., 2024), including participatory research and co-creation, seem to be most supportive of this right to be heard (Angelöw & Psouni, 2025; Coyne & Carter, 2018). Such approaches are characterized by the generation of knowledge and the creation of social change through collaborative efforts and innovative solutions, while fostering an inclusive and empowering environment for all people involved (Vargas et al., 2022; Vaughn & Jacquez, 2020). Given that young people are biologically, emotionally and developmentally capable of engaging beyond their families (Patton et al., 2016), it is essential to create opportunities for meaningfully involving them in processes of social design and civic action regarding issues affecting their lives and broader society (Kurth-Schai, 1988). The engagement of young people in research is not only ethically desirable from a rights-based and developmental perspective. Public policies that shape health and educational systems should consider the social, economic and cultural changes and challenges that young people face in contemporary societies (Patton et al., 2016). This is essential as to develop strategies that are relevant and sensitive to their needs and to address topics that impact the current and next generation of young people (Alves et al., 2022; Budin-Ljøsne et al., 2023; Zulu et al., 2018). Thus, also from a health-based and policy perspective, it is of utmost relevance to invest in the engagement of young people through collaborative research. Despite the different arguments in favour of engaging young people in collaborative research, there are some ethical concerns that may show up during the process. Over the past decades, there has been a vast amount of research conducted about the ethical implications of research with young people. An important ethical concern is that adults, including gatekeepers, make decisions about research participation on behalf of the young people (Schmid & Garrels, 2025). As collaborative research is often initiated by adults, issues of power imbalances are persistent (Coyne & Carter, 2018; Schmid & Garrels, 2025). Tokenistic practices, in which adult researchers hear the voices of young people but do not act upon them, are frequently reported and stem from adult dominance or adult-centrism in the research process (Morrow, 2008; Teixeira et al., 2021). Another ethical concern is that young people are not fully aware of the aims, methods and ethical implications of their engagement, which may lead to unclarities for the young people themselves (Coyne & Carter, 2018; Cullen & Walsh, 2020; Schmid & Garrels, 2025; Spriggs & Gillam, 2019). Although these concerns directly affect the young people engaged, current guidance on ethical practice and how to respond to ethical concerns within collaborative research is predominantly based on adult interpretations (Crane & Broome, 2017).

## Young people as moral actors in society

**Figure 1.**
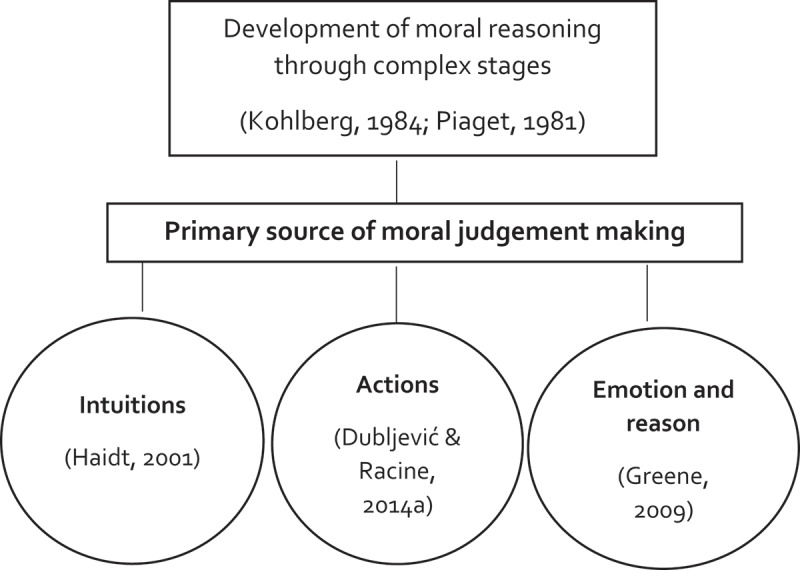
Theories of moral reasoning.

Young people are in a position of providing unique contributions (Patton et al., 2016). Earlier studies have demonstrated the ability of young people to voice their perspectives about societal issues, including social exclusion (discrimination), youth policies, violence, injustice and social innovation (Czibere & Paczári, 2021; Daiute & Fine, 2003; Müller, 2024). As they develop in hypothetical and abstract thinking and meta-cognitive processing, it is suggested that young people can be rightfully viewed as moral actors (i.e. individuals being able to making decisions based on a sense of right and wrong) within the research process (Bell, 2008; Patton et al., 2016). Moreover, insights from the fields of cognitive and moral development support the notion that young people are capable of thinking and reflecting about morality regarding their personal identity and values, obligations towards the self and others, and principles of justice and fairness in light of societal and interpersonal issues (Bajovic & Rizzo, 2021; Killen & Malti, 2015; Kohlberg, 1984; Nucci, 2001; Piaget, 1981; Rest, 1986; Smetana, 2006; Smetana & Turiel, 2003; Steinberg, 2005; Turiel, 2008). Morality can be understood as the way people treat each other, how they experience and deal with personal and others’ emotional experiences in the context of ethical and social conflict (Killen & Malti, 2015). Young people form an integral part of contemporary societies that are characterized by times of crisis and change (Malti et al., 2021; Patton et al., 2016). Considering the importance of nurturing responsible and inclusive citizens for peaceful, healthy societies and fostering positive relationships, it is essential to gain a comprehensive understanding of the mechanisms that drive moral growth for research on moral development in young people (Malti et al., 2021). Morality, justice and fairness are pivotal in every human culture and can be said to enable human society and social life (Yoder & Decety, 2018). From birth, human beings have the capacity to develop prosocial and moral behaviour. The specific culture and socialization process in which young people are born and develop influence the moral principles and duties they adhere to (Hamlin, 2015). Research on moral development, reasoning, and judgement in young people has made significant theoretical advances over the past decade (Malti et al., 2021). Early developmentalists such as Kohlberg (1984) and Piaget (1981) described moral development (including the development of reasoning and judgement) as a series of stages or patterns that become increasingly complex as children mature into adolescence. The role of conscious deliberation was central to this process (Malti et al., 2021; Warner et al., 2024), as young people eventually learn to engage with universal ethical principles, including justice. Haidt’s (2001) social intuitionist model emphasizes the role of fast, automatic intuitions as the primary source of moral judgements, rather than conscious deliberation (Pizarro & Bloom, 2003). The dual-process theory (Greene, 2023) takes a middle ground, suggesting that moral judgements are processed both emotionally (unconsciously) and rationally (consciously) (Warner et al., 2024). More recently, the ADC (Actions, Deeds, Consequences) model of moral judgement proposed by Dubljević and Racine (2014) argues that actions, deeds, and consequences are elicited intuitively and then evaluated to make a moral judgement (Białek et al., 2014). Figure 1 provides an overview of the different theories for moral reasoning and their connection. Generally, it can be stated that the moral reasoning of young people is influenced not only by their basic intellectual abilities (rational, conscious thinking) but also by their desires, motives, and interests (Steinberg, 2005).

## Moral reasoning and its role in shaping ethical collaborative research

Moral reasoning is an intrinsic part of the moral development of young people and can be explained as a cognitive activity characterized by several executive, emotional and motivational elements, in which transitions in thoughts in accordance with endorsed moral principles, such as justice, others’ welfare and rights, are processed (Jampol et al., 2010; Killen & Dahl, 2021). It allows young people to navigate the complex social world in which they live (Yoder & Decety, 2018). As such, it is suggested that moral reasoning is not only an essential part of human moral development, but also fundamental for societal change (Killen & Dahl, 2021). Moral reasoning changes during the transition from childhood to adulthood and is shaped by many components, including age, sex and ethnicity, and can differ per situation and context (Alves et al., 2022; Bajovic & Rizzo, 2021; Jampol et al., 2010). As young people form social connections and attachments in a broader social world (Barber & Erickson, 2001; Shao et al., 2024), they are positioned to develop the ability to understand and navigate competing interests in real-world interactions, while continuing to refine their moral reasoning and emotional regulation (Bajovic & Rizzo, 2021). The ability of young people to reason and act morally is influenced, in part, by their capacity to cognitively distinguish universal moral principles from social conventions (Bajovic & Rizzo, 2021; Turiel, 2008). This process occurs within a context where individual characteristics, along with the cultural and situational environment, shape their beliefs. As young people develop their capacity of moral reasoning, they increase their opportunities to exercise moral agency through making their own moral judgements in a complex and multidimensional context (Takagi & Saltzstein, 2023; Wynia et al., 2000), and through critiquing societal structures and providing arguments for unfair societal arrangements (Killen & Dahl, 2021). As such, their moral agency during collaborative research starts to take shape, facilitating the actualization of a more inclusive and ethical process.

Perceiving young people as moral actors in research and allowing their values and perspectives to shape the process does justice to their position as rights-bearing participants (Bell, 2008). In addition, Abebe and Bessell (2014) argue that ethical research is fundamentally rooted in recognizing young people as human beings by safeguarding their human rights and dignity, and acknowledging the influence of their values on interpersonal dynamics throughout the research process. This means a thoughtful consideration of young people’s moral reasoning is required, as this shapes their judgements about real-life moral dilemmas and ethical issues (Takagi & Saltzstein, 2023; Turiel, 2008). Moreover, research committed to incorporate the voices of young people, as is the case within collaborative research, should consider the biological, historical and social components, such as sex, class and ethnicity (Alves et al., 2022) that may shape their moral views. As the moral views of young people influence how they act and behave, it is essential to consider the heterogeneity of young people with regards to these views in order to establish an ethical process within the collaborative research (Carter, 2009; Crane & Broome, 2017; Hurst, 2008; Levine et al., 2004).

## The present study

Previous paragraphs have outlined the importance of moral reasoning for establishing an ethical practice within collaborative research. However, it is argued that when young people experience a lack of understanding of what influences their moral reasoning, this may impede their ability to make moral judgements (Bajovic & Rizzo, 2021). Incorporating meta-moral cognitive strategies (i.e., higher-order thinking processes) is instrumental in achieving an ethical practice as it may stimulate young people’s active awareness of moral reasoning (Bajovic & Rizzo, 2021). This goes hand in hand with a focus on the articulation of values and the recognition of local ethos, as well as a serious engagement with young people around the nature and meaning of ethics from a meta-moral cognitive approach (Abebe & Bessell, 2014; Bajovic & Rizzo, 2021). To date however, only few studies have incorporated the perspectives of young people themselves regarding ethical research and their experiences of, and solutions to ethical concerns (Crane & Broome, 2017; Denov & Shevell, 2021). This can be seen as a missed opportunity for enriching our current understanding of ethical practices within collaborative research, as young people are capable of providing creative, innovative and novel perspectives that otherwise remain unknown to the adult academic establishment (Patton et al., 2016). Engaging young people in a process whereby they acquire skills in moral reasoning and judgement may provide insights for academic researchers regarding their moral views, and thus unique needs, during collaborative research. The latter may potentially form an effective catalysator for positive social change, as is ultimately aimed for within such research. The current study, Youth, Ethics & Participation (YEP), aims to fill this knowledge gap by exploring the moral views and related reasoning of young people regarding the nature and meaning of ethics within collaborative research through the stimulation of meta-moral cognitive awareness. Moreover, YEP aims to develop methods that facilitate discussions and reflections (i.e. moral awareness) about ethical collaborative research. This may inspire future collaborative studies among young people to establish ethical practice. YEP is conducted as part of the Health CASCADE project that aims for developing trustworthy foundations and methodologies for collaborative research such as co-creation (Verloigne et al., 2023).

## Methods

### Study design and rationale

The YEP study adopted a qualitative phenomenological-educational approach (Alhazmi & Kaufmann, 2022; Stolz, 2023) with an action component (Meyer, 2000). Phenomenology can be perceived as an educational approach allowing researchers to engage in flexible activities that can describe and help to understand complex phenomena, such as various aspects of human social experience (Alhazmi & Kaufmann, 2022). Further, action research focuses on generating solutions and is purposively adopted for contributing to social change (Meyer, 2000). According to Hart and Bond (1995), four typologies of action research can be distinguished: experimental, organizational, professionalizing and empowering. Within the YEP study, the action component is used predominantly for empowering purposes, as its educative base was to raise consciousness about ethics in collaborative research and to inspire structural change towards more ethically sound processes within such research (Hart & Bond, 1995; Meyer, 2000). Hence, its strength lies in its focus on generating solutions to practical problems and its ability to empower practitioners through their engagement with research and action development activities (Meyer, 2000).

As YEP aimed at exploring the moral views about collaborative research through a phenomenological approach, young people were engaged in multiple activities that formed the methods for data collection. The educational purpose of YEP was to engage with young people around the nature and meaning of ethics in accordance with their moral reasoning (Abebe & Bessell, 2014). The study was therefore pre-designed by the academic researcher (RC) who designed and facilitated the group sessions. The sessions were mainly inspired by the Philosophical Ethics in Early Childhood (PEECh) programme (Arda Tuncdemir et al., 2022; Burroughs & Tuncdemir, 2017) and included some of the teaching strategies for developing meta-moral cognitive skills as outlined in the study by Bajovic and Rizzo (2021). PEECh is a combined social-emotional learning (SEL) and ethics education curriculum, developed in collaboration between ethics education and SEL researchers and early childhood educators. The original PEECh programme includes games, extension activities, and dialogue to support children in thinking about social and emotional dilemmas deeply, sharing their ideas, and creating solutions (Arda Tuncdemir et al., 2022). PEECh is found to increase children’s social – emotional competence and understanding of their own and others’ emotions. Further, Bajovic and Rizzo (2021) recommend four educational strategies for developing meta-moral cognitive skills. These include 1) the establishment of a safe environment in which young people honestly share their moral emotions, motivations and actions, 2) enabling a critical moral discourse in which young people discuss different moral issues and current events, 3) a connect and deconstruct strategy in which young people make connections between what they think, feel and act, and 4) moral reflection in which young people reflect on the consequences of choices made in the past and related feelings. The YEP sessions adopted elements from the PEECh programme in the study (e.g. games and dialogues) in addition to the strategies as proposed by Bajovic and Rizzo (2021) in the form of creative qualitative methodologies (see ‘Study elements’).

### Setting and participants

The project was conducted between April 2022 and November 2024 and was a collaboration between an academic researcher (RC) and a diverse group of young people. All sessions took place in Ghent, Flanders, Belgium. The city is the capital of the East-Flemish province with approximately 267.000 citizens. Ghent is known for its cultural, ethnic, linguistic, religious, social and economic diversity.

In the YEP study, young people are defined as those who are positioned between the transitional phase of childhood and adulthood with the age of 10 years through to age 24 years (United Nations Department of Economic and Social Affairs, n.d.). Three groups were established with a total of 23 young people (see Table 1). Previous involvement in collaborative research was desirable but not a requirement for involvement in YEP as difficulties were expected in finding only young people with such experience. The first group (n = 3) was formed through convenient recruitment as these were young people who had experience with a collaborative research on improving sleep behaviour (Vandendriessche et al., 2023). The second group (n = 15) and third group (n = 5) were formed through actively approaching and pitching YEP to a variety of youth organizations in Ghent. The groups were mixed in terms of age, sex, ethnic roots, education and religious affiliation or philosophical orientation. The sessions were organized at a location of preference of the young people.

**Table 1.** Characteristics of young people involved in YEP.

	Group A	Group B	Group C
Location of the sessions	University classroom	Educational cultural center and youth hub	Artistic cultural center
Previous experience with collaborative research	Yes	No	No
Number of young people	*n* = 3	*n* = 15	*n* = 5
Age (mean and range)	17 (Range: 17)	16 (Range: 14–18)	23 (Range: 21–26)
Sex	Young men (*n* = 2) Young women (*n* = 1)	Young men (*n* = 4) Young women (*n* = 11)	Young men (*n* = 1) Young women (*n* = 4)
Ethnic roots	Belgian (*n* = 3)	Moroccan (*n* = 8) Algerian (*n* = 4) Somali (*n* = 3)	Belgian (*n* = 2) Bosnian (*n* = 1) Tunisian (*n* = 1) Belgian/Senegalese (*n* = 1)
Education	Upper Secondary School	Upper Secondary School	Upper Secondary School
Religious affiliation or philosophical orientation	Atheism (*n* = 1), Christianity (*n* = 1), Islam (*n* = 1)	Islam (*n* = 15)	Atheism (*n* = 3), Pantheism (*n* = 1), Islam (*n* = 1)

### Study outline

Table 2 Provides an overview of the study outline. The sessions (*n* = 19) were divided into different parts, including: (a) informing and safe space creation (‘building rapport’), in combination with (b) acquiring theoretical knowledge about ethics and collaborative research (‘ethics education’), (c) engaging in discussions and reflections about ethical issues in life and collaborative research specifically (‘thought-provocation’) or (d) developing methods for improving the ethical practice of collaborative research (‘action’). Appendix A provides a descriptive overview of the methods used during each session and for each part. Further, each session was evaluated at the end which allowed for the improvement of some of those methods. For example, the presentation about the four levels of morality that was held as part of the ethics education (b) in the first group was changed into the ‘ethics tree’^1^ (see Appendix A), as young people found the presentation too abstract and theoretical. Throughout the project, the part on thought-provocation (c) was constantly enriched with new methods following the action element (d). Finally, young people from all groups were involved in a concluding session (*n* = 1) in which the most important findings were discussed, summarized and consolidated.

**Table 2.** The YEP study outline^2^.

	Group A (7 2 h- sessions across 4 months)		Group B (8 1 h-sessions across 1 month)		Group C (4 3 h- sessions across 4 consecutive days)	
Parts	Sessions	Methods (examples)	Sessions	Methods (examples)	Sessions	Methods (examples)
Building rapport (a)	1,2,3,4,5,6,7	Puzzle Playing football	1,2,8	Dilemma game Ball throwing	1,4	Dilemma game Cooking together
Ethics education (b)	2,3,4	Draw timeline of co-creation Four levels of morality	2,3,4,5	Thought shower ‘good’ and ‘bad’ Ethics tree	1	Thought shower ‘good’ and ‘bad’ Ethics tree
Thought-provocation (c)	2,3,4	Discussion about moral dilemmas Describing an excellent person	2,3,4,5	Street interviews Meaningful drawing	1,2	Discussion on how to decide on right and wrong
Action (d)	5,6	Thought shower ethics methods Dot voting	6,7,8	Thought shower ethics methods Dot voting	2,3	Thought shower ethics methods Dot voting

#### Building rapport

For young people to be able to make a conscious choice about being part of YEP, time was reserved for providing information about the project and asking related questions. In groups, the informed consent form was read aloud by a voluntary participant and questions could be asked about the objectives, aims and procedures of YEP. The facilitating academic researcher (RC) also repeatedly emphasized the non-compulsory attendance of the sessions. One of the essential elements was to incorporate a variety of games at the start of each session to build trust and rapport. As most young people never participated in a project focusing on ethics in collaborative research, the introductory games were often aimed at sensitization to related topics. Examples of introductory games were the ‘dilemma game’, ‘ball throwing’ and a puzzle (see Appendix A).

#### Ethics education

To increase the ethics vocabulary of the young people, the adult facilitator/academic researcher (RC) chose to share theoretical ethical concepts and statements that were subsequently discussed, such as explaining the philosophical concepts of ‘ethics’ and ‘morality’ (see Appendix A) to aid in capacity building. This acquisition of vocabulary was particularly important as young people were not acquainted with the theoretical concept of ethics in general and ethics in collaborative research in particular. Further, for the groups who did not have experience with collaborative research, extra attention was paid to explaining its general procedures and concepts. Ethics education took place through one-directional presentations, handing out sheets with ethical terms and concepts, and drawing a metaphorical ‘ethics tree’. As such, this session element was eventually designed to provide young people with the tools needed to engage in thought-provocation (c) and action (d).

#### Thought-provocation

Understanding young people’s perceptions on ethics in collaborative research required a set of methods aimed at facilitating discussions and critical thinking regarding ethics and ethical issues that may arise during the process. In line with the methodology of Community of Inquiry (CoI), the facilitating academic researcher (RC) engaged the young people in dialogues and interaction in a CoI that was focused on developing skills in communication and the co-construction of meaning (Arda Tuncdemir et al., 2022; Burroughs & Tuncdemir, 2017). The first methods performed in Group A were initiated by X. In Groups B and C, the methods used to incorporate the thought-provocation element (c) during the sessions, were developed by the young people themselves in the action phase of groups A and/or B (d). This resulted in an accumulation of methods that all contributed to a better understanding of their perspectives.

#### Action

Each group was involved in generating thought showers and developing methods. The action element (d) was consistent throughout the project and was enacted in a systematic manner for all groups. Through thought showers, pitching and dot voting, young people formulated a variety of methods that they believed would improve the ethical practice of collaborative research. During a closing session, young people collaboratively discussed and formulated the main characteristics of each of the methods that passed the selection procedure through the dot voting.

### Data collection and analysis

**Figure 2.**
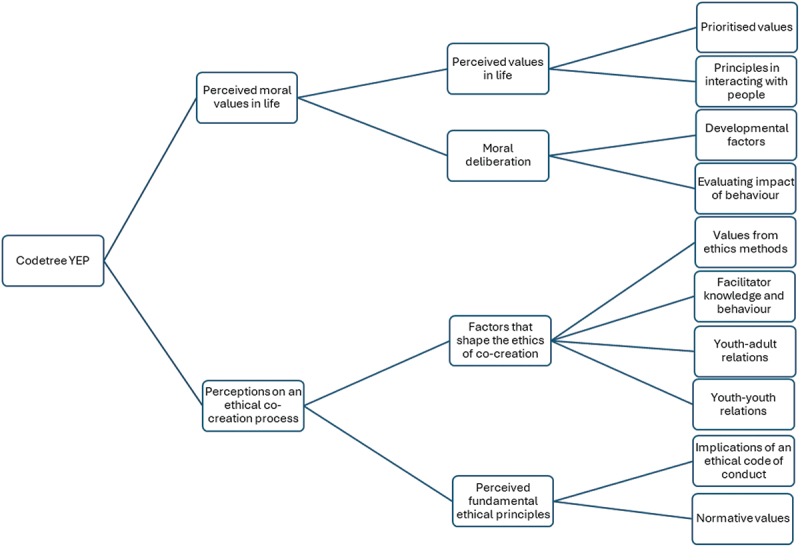
Preliminary codetree based on the double independent coding of three rich transcripts.

All 20 sessions (19 regular sessions + 1 closing session) were audio recorded and transcribed verbatim. Throughout the two-year project, the facilitating adult/academic researcher (RC) kept field notes and audio reflections following the sessions. The field notes and audio reflections helped further contextualizing the data. Data included the transcripts of the audiorecording of all sessions, drawings, paintings, and methods following the ethics education (b), thought provocation (c) and action phase (d). Data were synthesized using elements from thematic analysis (TA) and grounded theory (GT) (Braun & Clarke, 2019; Charmaz, 2014), including initial familiarization with the data (while developing sensitizing concepts), open coding and reviewing and defining main subjects through codetree development (see Figure 2). While identifying patterns in the data (TA), data was simultaneously analysed systematically and inductively with the aim of linking young people’s accounts to existing theories (GT) – thereby creating an explanatory theory of ethics in collaborative research through the eyes of young people. First, two researchers (RC and FF) acquainted themselves with the data by independently close-reading one rich transcript. The researchers subsequently developed a list of sensitizing concepts following a discussion. The concepts helped to create a general sense of reference and guidance in approaching the data (Blumer, 1954), but were not imposed on the data in a fixed way. Instead, we remained open to emergent categories and themes. The following sensitizing concepts were formulated: respect, group dynamics, solidarity, safe space, intersectionality, unicity, levels of knowledge, inclusivity, autonomy and boundaries, epistemic justice, racism, empowerment, multi-perspectivism and discrimination. In a second phase, RC and KM independently close-read three rich transcripts (one per group) with the sensitizing concepts in mind. The instruction was to perform open, inductive coding. The following questions guided the coding process: What are interesting topics that young people bring up? What are interesting quotes? What is your impression regarding the mindset of young people? What are things that they find disturbing/enriching? What core values do young people mention often? Examples of codes that emerged during this phase were: determining right and wrong, defining values, feelings of belonging, principles that determine behaviour, beauty of difference, serving general interest and humour as enlightenment. During a meeting, RC and KM discussed the codes and developed a preliminary codetree based on the analysis of the three rich transcripts (see Figure 2). The codetree was further approved by FF and MV. The remaining transcripts were analysed by RC based on the developed codetree. In Appendix B, a coding scheme explaining the different elements in the codetree can be found.

### Reflections on positionality

The sessions were facilitated by the first author (RC). She is a female doctoral researcher in possession of an MSc degree in Health Sciences, with a specialization in qualitative research, international public health, migration and human rights. She became interested in the topic of ethics in co-creation following several years of experience in conducting such research with young people. She had no relationship with the young people prior to the study commencement. During the sessions, RC participated in the introductory games that aimed for acquaintance with each other. RC made efforts to centralize the ideas of young people during the process, acting as a facilitator and guiding the discussions where needed. This included asking probing questions and providing examples. Co-authors who were involved in the data analysis (FF, KM) and day-to-day supervision (MV, FF, BD, TA) are a diverse group of experts in the field of health sciences and collaborative research. The process of data analysis can be characterized as a reflective and iterative process in which efforts were made to stay as close as possible to the wordings of young people. MG was involved during the broader supervision and is an expert in the field of (bio-)ethics. All decisions were made through extensive deliberation with the co-authors from the day-to-day supervision (MV, FF, BD, TA) with research integrity at its basis.

## Results

Analysis of the data collected during YEP resulted in eight subjects of interest: 1) meta-ethical beliefs and knowing what is morally right, 2) ideas about ethical collaborative research, 3) preparing for collaborative research, 4) defining common standards, 5) valuing ideas, 6) roles and responsibilities, 7) privacy and acknowledgement, and 8) methods to establish ethical collaborative research. In the following sections, a description per subject based on the wordings and moral views of young people is provided.

### Meta-ethical beliefs and knowing what is morally right

When discussing on what grounds one can know if something is morally right or wrong, or in other words the meta-ethical beliefs of young people, similar sources of moral direction were mentioned that could shape ones thinking about right and wrong. The influence of parents while growing up, or *what is learnt in childhood*”, was one of the most frequently mentioned source of moral direction, although young people broadened their understanding of upbringing throughout the discussions to observable behaviour of, and social interactions with, friends, family and society as a whole:

Yes, you can also see upbringing very broadly, but the people you mix with and such, they also shape your upbringing to a certain extent. I don’t know … and then also your friends, that they actually … No, that is actually development. People who are also responsible for your development and such, such as your friends, family, distant family, school, society, all those things will determine what is morally right. – Young man, Group A

Values, norms and images derived from conventional and social media were believed to shape their moral reasoning greatly: *‘I also think that social media plays a big role in that. Nowadays everyone is on TikTok and then you see that this or that is not okay to say or do. People dare to express their criticism about what is right and wrong more easily on social media.’* – Young man, Group B

Young people also mentioned the role of communities, school rules and regulations, beliefs and religions in shaping individual ideas about what is right and wrong: *‘I find it really hard to say if something is good or not good. One half can say it’s good. The other half can say it’s not good; that’s not our standards or values. And others can say it’s super good. It really depends on which communities you’re interacting with.’* – Young woman, Group C

Some young people found that social and cultural norms that are dominant in a particular place or time, or the social acceptability of human behaviour, cannot be used as a basis for knowing what is right or wrong in other times and contexts:

Because if you look at the past, it was socially acceptable to have slaves, but now not anymore. So were they bad then or was that just normal or good then. You know. It depends on how you see it. If you derive it from an objective source (i.e. God), then you suddenly know everything (everything becomes clear), then you know what is good and that remains (i.e. applicable in all contexts). But if you can’t get information from that objectivity, then you can’t really say what is good and bad. – Young man, Group B

Young people had different perceptions on what constitutes an objective source. Some mentioned God or religious revelation as an objective source. In the case of slavery, the young man mentioned God (Allah) as an objective source, referring to the prohibition of slavery within Islam. Other young people mentioned scientific evidence as an objective source, while a combination of these two sources was also suggested.

Although young people could easily mention sources for determining right and wrong, they found it difficult to think about how to determine what exactly constitutes ‘acting rightly’ during ethical difficulties or concerns. All groups however mentioned human intuition as an important influencing factor for determining how to act rightly. Young people did not use the same explanations for defining what their understanding of the concept intuition entails, but a common thread was that it refers to non-rational feelings based on previous life experiences and built-up moral values. Although young people mentioned that they rely on these intuitions for knowing what is morally right, they acknowledged that there are limitations:

And it could even be that you, for example, feel very right according to your intuition to do something, but that for someone else that was actually a less positive experience or something. So that also differs very much from which perspective and in which situation. – Young woman, Group C

Young people mentioned that the consequences or impact of one’s actions cannot be fully assessed prior to taking the actual action, as *‘you cannot be one hundred precent sure about how other people (will) feel about your actions’*, but there were some indicators according to them. Hurting someone or feeling hurt was an indicator of a bad or unethical action. Also, starting a conversation with others about the impact of your actions was perceived to be helpful in improving future behaviour. Some further mentioned that a good or morally right action *‘starts with an intention and has a spiritual component’* and that*‘acting rightly is what feels best for yourself’*. Despite these indicators, making choices was believed to always be a matter of accepting the choices one makes even though the consequences following these choices cannot be known in advance: ‘*It is actually about acceptance, also, for the choices you make.’* – Young woman, Group C

**Figure 3.**
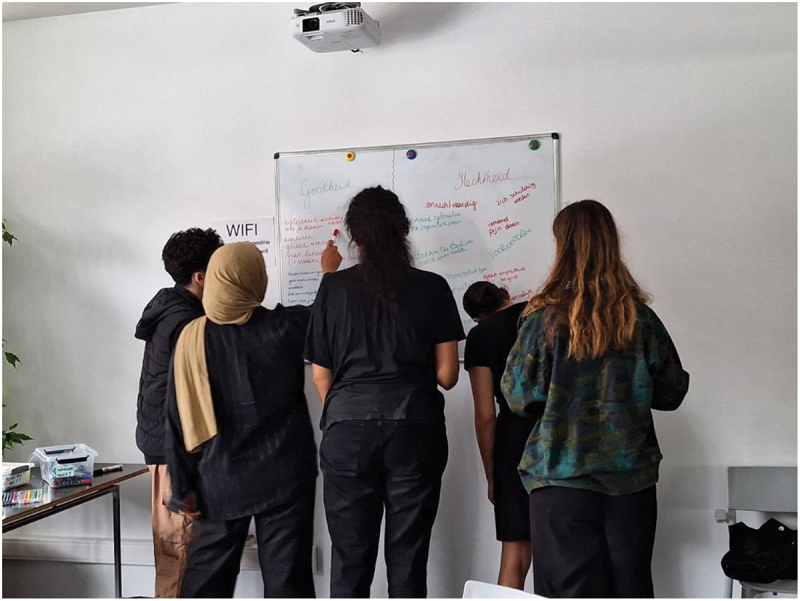
One of the groups writes down associations with ‘goodness’ and ‘badness’.

**Figure 4.**
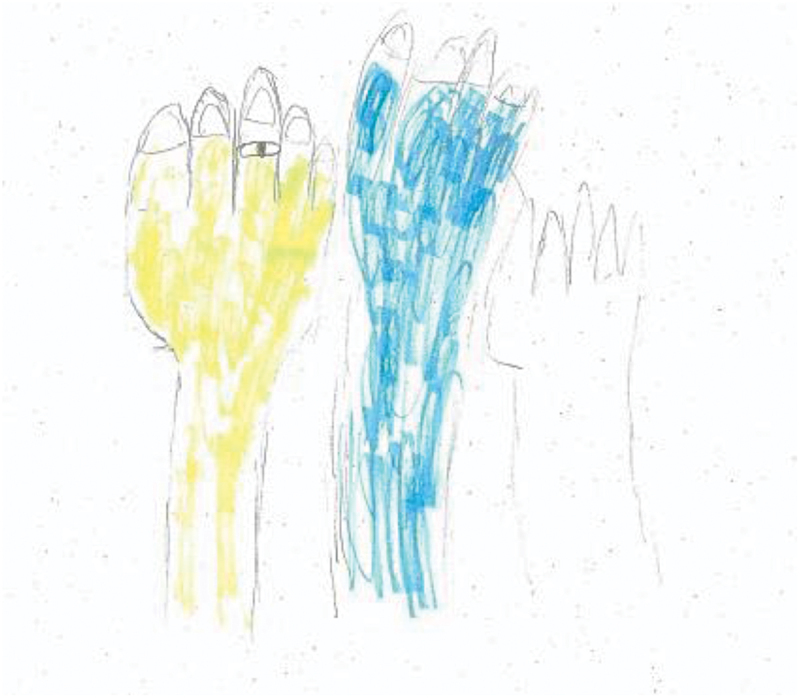
Drawing of humanity and non-discrimination – young man, group B.

Young people shared a variety of examples of ‘goodness’ during one of the sessions (see Figure 3). Goodness was explained as the morally right thing/attitude to do or have, and ‘badness’ as the morally wrong thing/attitude to do or have. Examples of goodness were: helping and saving people, accepting others and their views, being kind and merciful, being respectful, using proper, inclusive and respectful language, being optimistic, treating people equally, offering protection, being honest and compassionate, unconditional love, altruism, self-acceptation, respecting boundaries, sincerity, listening to yourself, daring to be yourself, respecting each other’s choices and having a reflective attitude. All groups mentioned having good intentions as an example of goodness. Examples of badness included: racism, prejudices, egoism, unwillingness to change, being unjust, greed, abuse of power, being inhumane, being a hypocrite or ‘fake’, manipulating others, hurting others and being an opportunist/using others to further your own life/goals, discrimination, cheating, narcissism, nihilism, arrogance, feelings of superiority, being naive, unfairness (inequity), stubborness, idleness, sabotaging others and disrespect. Racism and discrimination were mentioned by all groups. Figure 4 shows a drawing of humanity and non-discrimination.

**Figure 5.**
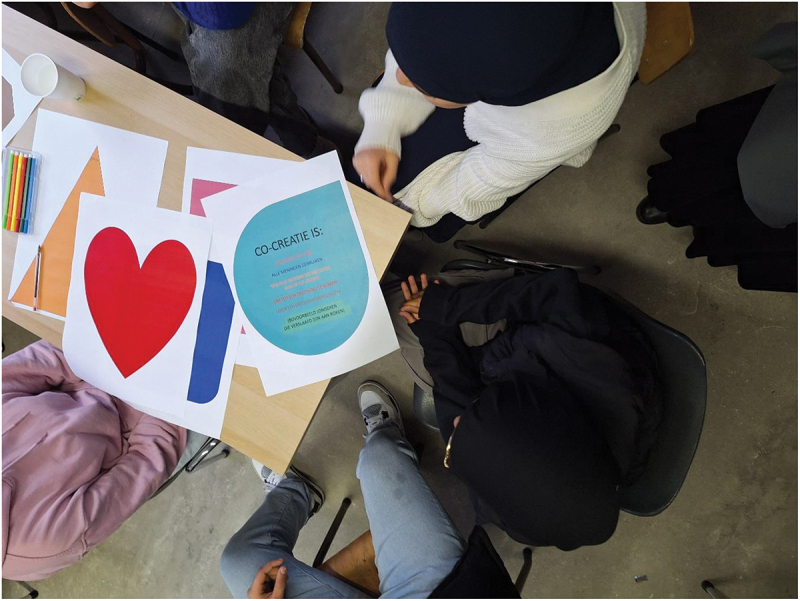
Mixed groups write down fundamental values for collaborative research during the closing session.

Young people agreed that ethical collaborative research means a form of collaboration in which respect should be prioritized. During the closing session, young people decided on a set of basic values (see Figure 5) that apply to all people involved in the research, including the young people and adults (see Table 3). Although the values apply to all those involved in the collaborative research, the upholding of some of these values could be placed under the responsibility of the facilitating adult or academic researcher. This is particularly applicable for the organization of fun activities, the facilitation of a safe space and the maintenance of a multi-disciplinary group. Relatedly, young people highlighted some boundaries, that should not be crossed to ensure an ethical collaborative research process. These include ‘*feeling really bad about participating*’ (e.g. emotional boundary), ‘*low trust in each other*’, ‘*adults who promise things that are not realistic*’, and ‘*not accepting each others’ visions (both adults and young people)*’. According to them, it would be justifiable to end the collaborative research or discontinue engagement if one of these boundaries are crossed.

**Table 3.** Fundamental values within collaborative research according to young people.

Value	Meaning
Respect and inclusiveness	Be open to other perspectives, listen, value diversity, develop mutual understanding.
Patience	Being committed to actively participate throughout the whole co-creation process.
Critical learning	Develop important and relevant insights about the topic, attentiveness during sessions, ask questions or pose ideas, advise others.
Commitment	Show enthusiasm and be actively involved.
Network	Maintain multi-disciplinary group of actors, actively seek contact with others.
Collaboration	Co-creation is all about working together towards a common goal.
Empathy	Be understanding and open to others.
Fun	Organise activities to facilitate interaction, building rapport and enjoying time together.
Safe space	Be able to share your opinion without fear.

Further, to visualize their ideas about what constitutes ethical collaborative research, young people were asked to make a painting. Table 4 presents some of the paintings accompanied by their respective interpretations.

**Table 4.** Paintings visualising ethical collaborative research.

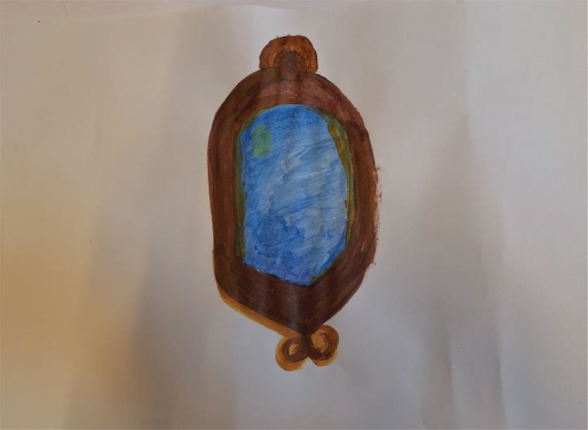	*“I have attempted to draw a mirror. In the broadest sense of what a mirror can do, on the one hand showing yourself and how you think about certain things, but also being a mirror to each other. Someone else can be the mirror. By listening to other perspectives, you can also think about how you think about something.”* – Young woman, Group C
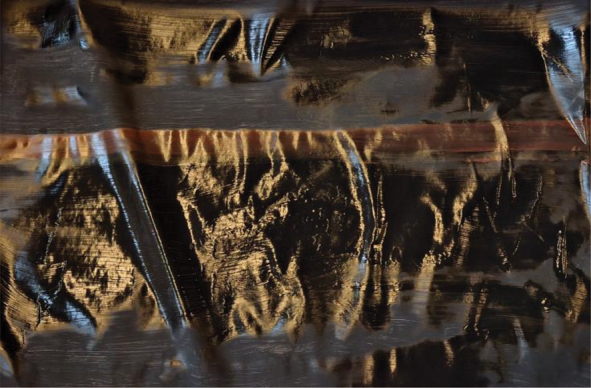	*“So what I think about* collaborative research *is that there is enough space in the black to feel like is this the right one, that there is space to think a lot about things that are not always seen quickly. But that it is ultimately important that we can stand on the same page together.”* – Young woman, Group C
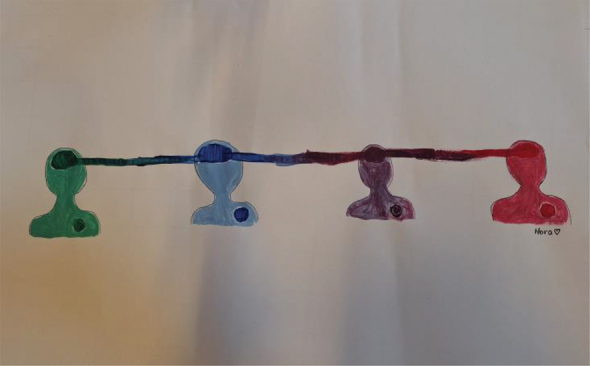	*“I have all people who are connected with their head. So head and heart have the same color, but the heart is not necessarily connected because they all stick to their own principles and intuition. But that they can find a kind of compromise in which they can be on the same page and still be connected without having to abandon their own gut feeling.”* – Young woman, Group C

#### Preparing for collaborative research

Two groups of young people mentioned that ethical collaborative research should start with formulating a morally acceptable research objective: *‘With certain goals you just can’t be okay if that is the main goal of your research […] Content-wise, what is that goal (is it ethical)? That you don’t go looking for the boundaries of what is okay (when formulating research objective).’* – Young man, Group A

Young people emphasized that initiating collaborative research requires careful consideration of which people to involve – while taking into account the ethical issues that may arise. For example, having a collaborative group with community members who live in extreme financial poverty together with for example rich academic researchers was considered as morally undesirable – as the academic researchers may lack sufficient sensitivity or empathy towards the community members’ context and life circumstances. In this specific example, young people mentioned *‘not wearing expensive clothes’* as a way to bridge the ‘social’ gap between the collaborators.

Varying other factors were mentioned that – according to young people – shaped the ‘ethical quality’ of the collaborative research. The role of language was considered extremely important by all groups. This did not only include choosing words carefully when sharing your opinion or being polite when talking to other collaborators, but also adapting to others in order to prevent miscommunication:

You have to adapt yourself to other values and standards if you can, otherwise miscommunication will arise. – Young man, Group B

Some disagreement among young people existed on how to address and respond to differences in knowledge, sex and individual values. Some found that collaborative research can be best initiated with people with the same level of education, upbringing or knowledge. Others stressed that heterogenous groups would be more inclusive of different perspectives and would lead to more thorough and nuanced outcomes. Some perceived differences between young men and women as a potential source of conflict, and stressed the importance of taking this into account, as is explained in the following quote:

I think the biggest conflict is between two different sexes, I think. That’s for sure, our vision will also be very different on different things. We haven’t always been the same in our lives, I mean … – Young man, Group A

#### Defining common standards

During discussions about the possibility of defining common standards through an ethical code for collaborative research, young people shared different considerations. Some stressed that such a code would be difficult to establish as a blueprint for all collaborative research initiatives, as ideas and situations may differ between groups of people. Further, being popular and being seen was perceived an important value for young people that needs to be considered when engaging with young people:

I think on the one hand that for example with young people that is also the case from puberty and such. That you also develop other standards through that – I think many people from our school find popularity very important, because with those large groups of friends, yes that is very important for some. – Young woman, Group A

Young people mentioned that establishing an ethical code based on shared values will be difficult, although there were some ‘*basic values such as respect and listening to other ideas*’ that were regarded as shared or universal values for ethical collaborative research. One of the young people mentioned that an ethical code should be partly based on a ‘*professional framework*’, with clear guidelines on how to participate in collaborative research. Another participant stressed that collaborative research in itself is not about building a unified set of ethical values, it is about truly listening and respecting diverse values and reaching consensus about how to proceed in an inclusive manner: *‘Collaborative research is about the inclusion of multiple values and truths, within collaborative research there cannot be one truth.’ –* Young man, Group B

A distinction was made between listening to ideas and embracing ideas, as ideas may undergo scrutiny by others involved in the collaborative research, and diminish in value throughout the process. Overall, having people, including academic researchers, with different or even opposing values on board was not seen as problematic, because the least that could happen is *‘having multiple ideas running parallel, and therefore multiple solutions to the issue at hand.’* – Young woman, Group A

#### Valuing ideas

Young people engaged in multiple discussions about how to value ideas that emerge during collaborative research. Objective ideas, based on observations, established sources, facts and scientific evidence, were generally perceived more valuable then ideas based on emotions, opinions and non-rational thinking. Some young people mentioned that there is a risk of *‘thinking in the wrong direction’* when this thinking is not based on the correct sources:

But then there is also a limit to how far you think differently, because at a certain point you can think so far that you think wrong. That something you say does not actually correspond to what is the case in reality. Should we then consider those ideas – well, those ideas of those people – to be equal to ideas that are okay? – Young man, Group A

During discussions about the value of acknowledging lived experiences and ‘being an expert concerning your own life’, young women tended to value lived experiences and view themselves as experts concerning their own lives, whereas boys tended to believe that people may not always know what is best for themselves: *‘Not everyone knows what is good and bad for themselves. Everyone, yes not everyone, many people think that what they do is good, while it is wrong.’* – Young man, Group B

#### Roles and responsibilities

Young people believed that the people involved in collaborative research are expected to have different positions and responsibilities. However, a prominent role was given to the facilitating adult. Young people mentioned several characteristics that the facilitating adultneeds to have. The most important one is being knowledgeable (having studied or being properly informed) about the topic that is explored/acted upon during the collaborative research. Other characteristics included being able to set up rules, manage the group, facilitate and bridge ideas and make decisions based on everyone’s input. One participant believed that there could also be multiple facilitating adults. Another participant posed the idea of *‘matching with the facilitating adult’*, or in other words, there has to be a positive attitude from both the young people and the facilitating adult to work together, as*‘you will spend many hours together’.*

#### Privacy and acknowledgment

Another discussion was about the adherence to rules of privacy. Some young people mentioned that if someone is extremely attached to personal privacy, engagement in collaborative research will be difficult as one will share personal ideas and stories. Regarding acknowledgement, they indicated that this was not only relevant for young people who participate, but also organizations who give money to fund such research. Young people disagreed on how to acknowledge the contribution of young people. One of the complicating factors according to them is that the contribution of involved young people is not always continuous as some may be very active in the beginning, and eventually not show up for the remainder of the project. Some young people did not understand the added value of reporting names in the final product, because *‘who’s going to go all the way down and then in that tiny little print of ah yeah… they contributed to that topic.’* – Young man, Group A. Further as this young woman (Group B) commented: *‘Yes, thanks to all the employees, then it sounds like you are lower or like you have done less than the leader (i.e. facilitator adult/academic researcher).’*

Others did attach value to naming the participants, so that their contributions will be recognized: *‘Yes, you go through something and then all the names are just there. Like in a movie at the end.’* – Young man, Group B. Additionally, following one of the discussions about copyright, young people found that there should be copyright on the ideas of young people, as there may be other people who are more capable (have resources) to execute their ideas quicker and then *‘walk away with all the credit, and then the rest (i.e. the young people) supposedly did nothing.’* – Young man, Group B

### Methods to establish ethical collaborative research

Young people and the facilitating academic researcher (RC) co-developed 65 ideas for methods to reflect on the ethical process of collaborative research. Seventeen of these methods were selected by the young people after consecutive dot voting in all groups. During the closing session, young people of all three groups were asked to formulate key characteristics of these methods in order to understand what ethical aspects are highlighted in these methods. Table 5 provides an overview of the selected methods, their description and characterizing elements as formulated by young people. One of the most dominant characteristics was that of playful acquaintance. Young people attached great value to getting to know each other in a fun way without focusing too much on heavy ethical issues at the start. Examples of methods that centralize this playful acquaintance include ‘*Roleplay*’ and ‘*Head and Catch*’. At the same time, young people mentioned that one should not share ‘*too private information*’ during the acquaintance. Rather, the methods should be introductory and instrumental for building a sphere of trust. Empathy through listening, knowledge generation and collaboration was another important characterizing element. This was reflected through several methods, including *‘Cultural Thinking’, ‘Dilemma Game’, ‘Stereotypes’, ‘Ethics Lessons’, ‘Ethics Dialogue’, ‘VR Glasses’* and *‘Roleplay’*. Lastly, young people found it necessary to incorporate methods that help increase insight about ethical boundaries in order to recognize and then anticipate what could *‘go wrong’* during the process. Methods that reflect these characteristics are *‘The Bad Researcher’*, *‘The Excellent Person’*, *‘Guess the Principle’* and *‘Ethics Interviewing’* – as they have in common that they help those involved in the collaborative research understand what moral views each person upholds.

**Table 5.** Overview of the selected methods.

Method	Invented by	Description	Characterising element
The Excellent Person	RC	Describe the most ethical character traits possible to understand ideal manners.	Social, empathetic, communicative, insight, open, ideals, non-judgemental, listening, communication
Ethics Dialogue	RC	Keep the conversation alive about ethical issue that have arisen or that may arise.	Knowledge, critique, non-judgemental, communication
Ethics Interviewing	Young people (Group A)	Get an idea about what others understand of ethics.	Insight, critical thinking, knowledge, listening
The Bad Researcher	Young people (Group A)	Make a portrait of bad adult (academic researcher) behavioural conduct and discuss afterwards.	Shape and voice opinion, being involved, insight, critical thinking
Line Game	Young people (Group A)	Present controversial ethical statements and make people vote pro or contra by standing in front or back in the line and also discuss afterwards.	Shape and voice opinion, being involved
Stereotypes	Young people (Group A, B, C)	Unravel prejudical convictions that exist within the group about each other and challenge these prejudices.	Empathise, knowledge
Meaningful Drawing/Meaningful Movement	Young people (Group A)	Draw or perform a movement that reflects an important value to you.	Norms and values, culture, creativity
Ethics Lessons	RC	Provide plain explanation of what ethics is and how ethical issues can be recognised.	Knowledge
Memorygame	Young people (Group B)	Link norms to their connected values regarding ethics in co-creation.	Collaborating, insight
Cultural Thinking	Young people (Group B)	Reason from a different worldview, conviction or societal position about a controversial topic.	Diversity, curiosity, knowledge
VR Glasses	Young people (Group C)	Visualise ethical dilemmas or situations, discuss these and find a solution together.	Discover, empathise
Dilemma Game	Young people (Group C)	Playful game for trying to solve ethical issues using cups and ping pong balls.	Communication, taking critical decisions
Guess the Principle	RC	Get acquainted with personal fundamental life principles through guessing what principle belongs to whom.	Communication, insight
Cooking Together	Young people (Group C)	Prepare a meal while sharing personal stories and views.	Having fun, acquaintance, being together, insight
Roleplay	Young people (Group A, B, C)	Mimic an ethically challenging situation and discuss afterwards.	Playful, icebreaker, empathy
Head and Catch	Young people (Group B)	Playful game to orientate on the topic of ethics and participation using your head and a ball.	Learning, sharing knowledge, playful

## Discussion

The current study aimed at engaging young people around the nature and meaning of ethics within collaborative research. Young people shared their perspectives on the meaning of ethical collaborative research, while showing their interest and ability of moral reasoning through the formulation of arguments explaining their position on a variety of ethically salient issues within collaborative research. Further, young people demonstrated their collective creativity in sharing a wide spectrum of ideas for methods to improve the ethical practice of collaborative research. As such, the current study reveals multi-faceted aspects to ethical collaborative research as identified by young people that will be described in the next paragraphs.

Young people mentioned different sources that shaped moral reasoning, and emphasized the dominant role of societal norms in shaping what can be viewed as morally right or wrong. It seems that social norms and personal intuition often underly moral judgements. The findings suggest however, that social norms and personal intuition are not sufficient to fully grasp morality, as young people explained that sometimes an external, independent source is needed. For controversial topics that are not easily solved through social norms and personal intuition, young people tend to turn to external sources for moral judgement (scientific knowledge, religious revelation – both considered as ‘objective’ sources). As this finding points to the limitations of human intuition, it can be stated that the social intuitionist model that emphasizes the role of fast, automatic intuitions as the primary source of moral judgement (Haidt, 2001) lacks in providing a comprehensive clarification for the accounts of young people. Moreover, according to young people, behaviour can be evaluated through assessing the consequences of that behaviour on others. They also emphasized the role of intentionality prior to behaving and the importance of identifying the benefits of a certain behaviour before acting. Therefore, a better clarification of the accounts of young people lies in a combination of the dual-process theory (Greene, 2023) and the ADC model of moral judgement (Dubljević & Racine, 2014) that stress the importance of both conscious and unconscious reasoning and the role of behavioural consequences for shaping moral judgements. The findings further suggest that the meta-ethical beliefs (i.e. beliefs on the nature, scope and meaning of ethical judgements, for example whether objective ethical values exist or not) differ among young people. Exploring these beliefs through the ideas for methods developed by young people may enrich academic researchers’ understanding of young people’s perspectives on what constitutes an ethical difficulty and what pathways they might consider for handling that difficulty. This is particularly relevant as judgements and explanations are based on meta-ethical beliefs which in turn shape our inferences about the ethical commitment and judgements of others (Lewry et al., 2024). Further, as the lived reality and epistemologies of people involved in collaborative research may differ, it is of importance to understand these to enhance collaboration within and to develop an ethics of practice (Bainbridge et al., 2013). Examples of methods that may contribute to a better understanding of these beliefs include ‘*Meaningful Drawing/Movement*’ and ‘*Cultural Thinking*’ as they both focus on the expression of personal convictions, norms and values.

The set of values that young people agreed upon as being fundamental for establishing ethical collaborative research show similarities with previous attempts of developing ethical guidance for such research. To illustrate, both emphasize the importance of inclusivity, collective action and active learning (Aussems et al., 2024; International Collaboration for Participatory Health Research [ICPHR], 2022). The importance of active or critical learning was expressed through the methods of ‘*Ethics Interviewing*’, ‘*Ethics Dialogues*’ and ‘*Ethics Lessons*’, as they all focused on becoming more knowledgeable about ethics in everyday life. Moreover, being caring and empathetic were both addressed by young people in our study as by earlier studies (Dedding et al., 2023; Groot & Abma, 2022). Methods that, according to young people, may contribute to a caring and empathetic environment include ‘*Stereotypes*’, ‘*Meaningful Drawing/Movement*’, ‘*VR Glasses*’ and ‘*Roleplay*’. Further, the substantive values they formulated, such as critical learning, show great overlap with theories of empowerment and positive youth development that prioritize critical consciousness for the betterment of ones life (Chrifou et al., 2024; Messiha et al., 2023). Additionally, young people mentioned values that are potentially overlooked or underestimated by current ethical guidance – such as patience and the incorporation of fun activities as a means to garner rapport and facilitate interaction and mutual connection. The suggested methods of ‘*Cooking Together*’ and ‘*Head and Catch*’ illustrate this need for mutual connection, as they primarily focus on getting to know each other. In general, young people agreed that finding a common ground with the principle of respect at its basis was the primary ethical principle for engaging in collaborative research. They attached great value to not overpromising and to being honest about expectations. Using appropriate language, being reflective and communicating openly were perceived as very important in this regard. This is relevant as earlier studies have pointed out that the risk of tokenism within collaborative research is still prevalent as a result of the power asymmetries between adults and young people (Arunkumar et al., 2018).

All groups mentioned racism and discrimination as morally wrong actions, and having good intentions as a morally right action. This may indicate that young people find it important to uphold inclusive practices during collaborative research and to choose a morally praiseworthy purpose for that research while upholding transparency. Young people agreed about respect as a foundation for ethical collaborative research. They further seem to attribute more responsibility to the adults facilitating the collaborative research to engage in proper moral reasoning, as they are expected to make morally right decisions during the research process. Although young people themselves want to partake in decision-making as well, they seem to ‘outsource’ this activity for an important part to the adult facilitator. Young people considered equity to be an important principle for ethical collaborative research, as they emphasized the need for properly ‘matching’ people. However, there were some differences with regards to how to respond to differences in knowledge, sex and individual values. Relatedly, establishing common ethical standards was not evident. According to Edgar Morin (2001), there are several obstacles for establishing shared ethical frameworks. These include ‘the polysemy of notions’, which means that concepts can mean different things based on what is learned and acquired. Importantly, young people want to be seen and heard; they seek recognition and validation, not just through the acknowledgement of their perspectives, but through actively incorporating and acting upon them in decision-making and actions, as is aimed for within collaborative research (Vaughn & Jacquez, 2020).

Making well-considered choices throughout the collaborative research was generally perceived a good ethical practice. This was illustrated by the fact that young people mentioned the formulation of an ethical (research) objective from the start, and that careful consideration should be given to which people to include, or, as the young people formulated, ‘*how they would match*’. There were some differences between young women and young men regarding the issue of idea generation and appreciation. Whereas young men tended to prefer the inclusion of ideas based on rational arguments and ‘objective sources’, young women were more prone to appreciate lived experiences as a valid source of generating ideas. Young men further argued that the risk of thinking in the ‘wrong way’ and the distinction between ‘hearing and embracing ideas’ supports the need for including ideas based on rational arguments. According to them, the validity of ideas should be ‘tested’ throughout the collaborative research. Moreover, they stated that one does not always know what is best for oneself, as they indicated that there should be an external source (such as scientific evidence) to guide ones choices. Although gender differences in moral reasoning have been noticed in an earlier study conducted by Takagi and Saltzstein (2023), where the authors state they there may gender-specific paths for developing moral thinking, the current study lacks data to fully compare potential differences. Further, it is unclear to what extent trusting a preferred knowledge source is contrary to the notion of upholding epistemic justice within collaborative research. Epistemic justice is explained as the equal appreciation of academic knowledge, practical wisdom, experiential expertise, and artistic knowledge (Groot et al., 2023). According to this notion, there is no such thing as a monopoly on knowledge that can claim objectivity and neutrality, to define the world around us (Groot et al., 2023).

As previously mentioned, young people attributed more responsibility to the facilitating adult than to the young people participating. According to them, the facilitating adult plays a pivotal role in the whole process and should be knowledgeable and able to manage diverse groups. Relatedly, the method of ‘*The Bad Researcher*’ shows that young people find it important to be aware of unfavourable facilitating behaviour. Moreover, this finding indicates that young people appreciate the establishment of order and opportunities for learning. According to them, this can best be realized through a skilled facilitating adult. This perspective is also underpinned by one of the young people who stated that professionality and clear guidance (mainly from the facilitating adult) should form the basis of a moral framework within the collaborative research. This is in line with Wong’s typology of youth participation that states that both adults and young people engage in a process of co-learning with the support of adults to achieve an optimal participatory environment (Wong et al., 2010). Lastly, young people were very strict and direct concerning a necessity to maintain specific boundaries during collaborative research. They did not tolerate the potential crossing of these boundaries, and emphasized the necessity of clear consequences if such boundaries were crossed. Young people found the methods of ‘*The Excellent Person*’ and ‘*Guess the Principle*’ suitable for understanding boundaries. Through the formulation of excellent character traits and important individual principles, a comprehensive picture of ideals and ideal behaviour can be created. This may contribute to a better recognition of situations where (personal) boundaries are crossed. Respecting these boundaries however may not always be realistic and at times difficult to implement in real-world research settings, as different complexities may show up – including complexities related to fulfiling project goals and reaching funding terms (Oliver et al., 2019).

### Educational reflections

Although young people demonstrated their capability of being moral actors through their moral reasoning on a variety of subjects relevant to collaborative research, it is worthwhile to reflect on the educational process. YEP aimed to engage young people around the nature and meaning of ethics through accessible ways that sparked both creative and critical thinking while providing a safe and playful environment – all of which may have stimulated the young people to enthusiastically participate and share their perspectives (Lipman, 1995). Most of the young people who participated in YEP had limited knowledge about ethics and did not participate in previous collaborative research for health promotion purposes within an academic setting. However, the findings suggest that earlier engagement in collaborative research is not a requirement for being able to reflect on an ethical process as young people shared similar views. Future research should explore to what extent the perspectives of young people regarding ethical collaborative research is shaped by earlier engagement in such research. Moreover, some studies suggest that collaborative research becomes increasingly politicized (Turnhout et al., 2020), potentially leading to a dominant focus on policy issues with no room for philosophical reflection and creativity (Franck & Osbeck, 2017). However, within YEP, the focus was to integrate this philosophical reflection and creativity as much as possible while discussing relational ethical issues during the ethics education. Another relevant reflection might be with regards to the risk of conformity to dominant standards that shape moral reasoning. In his work on the ‘moral audience’, Day (1991) describes moral actions as ‘a function of the audience to which they are played, just as moral stories are a function of the audience to which they are told’. The author argues that moral judgements are influenced by an interdeterminacy of voices, other persons, dialogues, personal narratives, socially given narrative structures, and other social factors (Day, 1991; Tappan, 1992). This interdeterminancy within YEP may have occurred as a result of several factors, including the pre-defined structure of the ethics education, the dynamics between the adult facilitator/academic researcher and the young people, and the differences in experience and knowledge of ethics in collaborative research among the young participants. Moreover, the age range (14–26) within YEP may suggest developmental differences in the ability of moral reasoning. For example, it is observed that ‘older’ participants in YEP showed greater skill in thinking about and formulating arguments regarding ethical issues within collaborative research. In addition, during the action phase, it is observed that the ‘younger’ participants co-developed more playful methods (such as ‘*Head and Catch*’) compared to the methods co-developed by ‘older’ participants (for example ‘*Stereotypes*’). However, it is recommended that future research explores these age differences more thoroughly.

### Methodological strengths and limitations

We managed to include a diverse group of young people with differences in sex, age, ethnicity, and religious background, which may be perceived as a methodological strength. Further, as the sessions were based on different elements based on earlier studies, this may have strengthened the facilitation of capacity building and the inclusion of various perspectives. A methodological limitation of the study might be the differences in acquaintance among young people with regards to the concepts of collaborative research and/or ethics. Additionally, the lack of consistent terminology regarding the concepts of collaborative research and ethics within literature might have complicated the ethics education. These two factors may have influenced the perspectives of young people on how collaborative research is perceived, especially with regards to roles and responsibilities.

### Implications for practice

The current study indicates that the engagement of young people in collaborative research is a potential source for instigating social change as they are capable of challenging established notions and engage in moral reasoning (Ho et al., 2015; Killen & Dahl, 2021). For future collaborative research endeavours, it might be worthwhile to further elicit and amplify young people’s voices regarding complex contemporary issues, including systematic inequality, mental health and human rights. Adult facilitators need to embrace their role and not devalue themselves by excessively diminishing hierarchical roles, as young people shared that hierarchy might be desirable when framed and acted upon in appropriate ways. This might be realized when shared decision-making is performed in collaboration with young people (Wong et al., 2010), and points to the need for structure and professionality in order to establish sufficient trust and a safe space for each individual within the group. Hence, there might be a need to redefine the notions of equality and equal relations within collaborative research, as it currently potentially disregards the unique contribution each person has in complementing each other and forming a collective within the process.

The ethics methods young people chose were mostly playful, focusing on getting to know each other, building understanding and empathy, and becoming aware of rights and duties during the process. The co-developed ethics methods provide a deeper understanding of the values that young people find worthwhile to protect during collaborative research. Developing insight, critical thinking, and communication are some examples of values that young people repeatedly mentioned. The ethics methods may serve as ways to enhance interaction, mutual understanding and create a safe atmosphere where ethical conduct can be inspired and stimulated, while potentially leading to increased moral awareness. The ethics methods should be evaluated in the future, to assess their potential of improving the collaborative research process from an ethical perspective. Moreover, future research should assess to what extent the ethics methods can improve existing ethical guidance on collaborative research with young people – for example by putting more emphasis on playfulness, acquaintance and developing commitment (patience). In Appendix C, a summary of the practical value per ethics method can be found.

## Conclusion

The YEP study aimed at engaging young people around the nature and meaning of ethics within collaborative research. The study shows that young people demonstrated interest and engagement with ethical concepts and were able to voice their opinions about matters beyond the standard procedural ethical issues – such as issues of assent and dissent (Crane & Broome, 2017) – to subjects that touch on principles and considerations within collaborative research, including epistemic justice, knowledge production, roles and responsibilities. This study also shows that, when involving young people in collaborative research, it is important to get to know them better, both their moral views, their existing knowledge on relevant topics within collaborative research and their meta-ethical beliefs. This might contribute to a deepened understanding of ethical collaborative research and to a further inclusion of young people’s voices in research, which in turn may help actualizing the empowerment of the young people themselves and their direct (family and friends) and broader (school and society) environment (Chrifou et al., 2024).

## Data Availability

The complete dataset as stored in MAXQDA are available from the corresponding author upon reasonable request.

## Notes

1. Metaphorical tree explaining the leaves as ones actions, the core as the intentions and the roots as ones worldview.

2. Detailed descriptions of the applied methods are provided in Appendix A.

## Appendix A: Overview methods used in the YEP sessions for part b and c

**Table:**

Group A		Group B		Group C	
Methods	Description	Methods	Description	Methods	Description
**Co-creation timeline**	Think in groups about the different phases of co-creation.	**Thought shower on goodness and badness**	Writedown your associations of goodness and badness.	**Thought shower about ethics and co-creation**	Write down your associations of co-creation and ethics.
**Moral dilemmas**	Discussing common moral dilemmas in co-creation.	**Ethics tree**	Metaphorical tree explaining the leaves as ones actions, the core as the intentions and the roots as ones worldview.	**Discussion meta-ethics**	Share your ideas about how you decide what is good and what is bad?
**Four levels of morality**	Presentation about the levels of morality according to Veatch and Guidry-Grimes (2007).	**Street interviews**	Interviewing random people on the street about their associations with ‘good’ and ‘bad’.	**Ethics tree**	Metaphorical tree explaining the leaves as ones actions, the core as the intentions and the roots as ones worldview.
**Paint your values**	Think of colours that you associate with co-creation. Inspired by the colour theory (Wabisabi).	**Meaningful drawing**	Make a drawing that symbolizes the most important values in life to you.	**Design your own co-creation**	Think of a co-creation in a group or individually related to health promotion, based on existing knowledge.
**Discussion**	Discussing the level of meta-ethics according to Veatch and Guidry-Grimes (2007).	**Design your own co-creation**	Think of a co-creation in a group or individually related to health promotion, based on existing knowledge.	**Painting**	Make a painting that visualizes an ethical co-creation.
**The excellent person**	Describe the character traits of the most (existing or non-existing) person you can think of.	**Challenging statements**	Researcher presents statements about ethically relevant issues and adolescents vote pro or contra.		
**Locations of co-creation**	Where does co-creation takes place?				
**Discussion**	Discussing the ethical boundaries of co-creation.				
**Think of a saying for co-creation**	Think of the core principle of co-creation and formulate that in one saying.				

## Appendix B: Coding scheme

**Table:**

**THEME 1/Perceived moral values in life**	
*Perceived values in life*: What values are important in day-to-day interactions and situations?	*Prioritised values*: Which values are given priority over others when dealing with a certain dilemma? *Principles in interacting with people*: What are important principles to uphold when interacting with people?
*Moral deliberation/reasoning*: What are reasoning patterns of youth when dealing with ethical situations?	*Developmental factors*: What factors shape the reasoning patters implicitly or explicitly? *Evaluating impact of behaviour*: How do youth evaluatie whether they made the right or wrong choice when dealing with a certain situation?
**THEME 2/Perceptions on an ethical co-creation process**	
*Factors that shape the ethics of co-creation*: What factors influence the ethical character of the co-creation process?	*Values from ethics methods*: What values can we derive from the methods developed during YEP to contribute to an ethical co-creation process? *Facilitator knowledge and behaviour*: What are desired characters of the (research) facilitator that help realizing an ethical co-creation process? *Youth-adults relations*: How are youth-adult relations influencing the ethical process of co-creation? *Youth-youth relations*: How are youth-youth relations influencing the ethical process of co-creation?
Perceived fundamental ethical principles	*Implications of an ethical code of conduct*: What are the pros and cons of instigating an formalized ethical code of conduct for the co-creation process? *Normative values*: What normative values should be upheld to ensure an ethical co-creation process?

## Appendix C: Overview of practical value per ethics method

**Table:**

Method	Description	Characterising element	Practical value
The Excellent Person	Describe the most ethical character traits possible to understand ideal manners.	Social, empathetic, communicative, insight, open, ideals, non-judgemental, listening, communication	This method can be used to understand the perceptions about ideal behaviour.
Ethics Dialogue	Keep the conversation alive about ethical issue that have arisen or that may arise.	Knowledge, critique, non-judgemental, communication	This method can be used to exchange ideas about ethical statements or issues.
Ethics Interviewing	Get an idea about what others understand of ethics.	Insight, critical thinking, knowledge, listening	This method can be used as an introduction to ethics and to develop initial ideas about the meaning of ethics.
The Bad Researcher	Make a portrait of bad adult (academic researcher) behavioural conduct and discuss afterwards.	Shape and voice opinion, being involved, insight, critical thinking	This method can be used to initiate discussion about boundaries, rules and rights.
Line Game	Present controversial ethical statements and make people vote pro or contra by standing in front or back in the line and also discuss afterwards.	Shape and voice opinion, being involved	This method can be used to initiate discussion about controversial ethical statements and to explore the different perceptions in the group.
Stereotypes	Unravel prejudical convictions that exist within the group about each other and challenge these prejudices.	Empathise, knowledge	This method can be used to address and potentially dismantle existing stereotypes.
Meaningful Drawing/Meaningful Movement	Draw or perform a movement that reflects an important value to you.	Norms and values, culture, creativity	This method can be used to explore important values that exist in the group.
Ethics Lessons	Provide plain explanation of what ethics is and how ethical issues can be recognized.	Knowledge	This method can be used to educate the young participants about ethics.
Memorygame	Link norms to their connected values regarding ethics in co-creation.	Collaborating, insight	This method can be used to playfully introduce young people to the ethical concepts of norms and values.
Cultural Thinking	Reason from a different worldview, conviction or societal position about a controversial topic.	Diversity, curiosity, knowledge	This method can be used to broaden the perspectives of participants about controversial topics.
VR Glasses	Visualise ethical dilemmas or situations, discuss these and find a solution together.	Discover, empathize	This method can be used to initiate discussion about real-life situations and to develop empathy for ethical difficulties.
Dilemma Game	Playful game for trying to solve ethical issues using cups and ping pong balls.	Communication, taking critical decisions	This method can be used to understand intuitional solutions to ethical issues.
Guess the Principle	Get acquainted with personal fundamental life principles through guessing what principle belongs to whom.	Communication, insight	This method can be used to explore important ethical values and principles of young people in the group.
Cooking Together	Prepare a meal while sharing personal stories and views.	Having fun, acquaintance, being together, insight	This method can be used to create a safe space.
Roleplay	Mimic an ethically challenging situation and discuss afterwards.	Playful, icebreaker, empathy	This method can be used to improve moral judgements in real-life ethically challenging situations.
Head and Catch	Playful game to orientate on the topic of ethics and participation using your head and a ball.	Learning, sharing knowledge, playful	This method can be used to understand existing knowledge on the concepts of ethics and participation.
